# Design of intelligent module design for humanoid translation robot by combining the deep learning with blockchain technology

**DOI:** 10.1038/s41598-023-31053-5

**Published:** 2023-03-09

**Authors:** Fan Yang, Jie Deng

**Affiliations:** 1 School of Foreign Languages, Fuzhou University of International Studies and Trade, Fuzhou, 350202 China; 2 Graduate School, University of Perpetual Help System DALTA, 1740 Las Pinas, Philippines; 3 Rockchip Electronics Co., Ltd., Fuzhou, 350007 China

**Keywords:** Computer science, Information technology

## Abstract

To accelerate the deep application of deep learning in text data processing, an English statistical translation system is established and applied to the question answering of humanoid robot. Firstly, the model of machine translation based on recursive neural network is implemented. A crawler system is established to collect English movie subtitle data. On this basis, an English subtitle translation system is designed. Then, combined with sentence embedding technology, the Particle Swarm Optimization (PSO) algorithm of meta-heuristic algorithm is adopted to locate the defects of translation software. A translation robot automatic question and answer interactive module is constructed. Additionally, the hybrid recommendation mechanism based on personalized learning is built using blockchain technology. Finally, the performance of translation model and software defect location model is evaluated. The results show that the Recurrent Neural Network (RNN) embedding algorithm has certain effect of word clustering. RNN embedded model has a strong ability to process short sentences. The strongest translated sentences are between 11 and 39 words long, while the weakest translated sentences are between 71 and 79 words long. Therefore, the model must strengthen the processing of long sentences, especially character—level input. The average sentence length is much longer than word-level input. The model based on PSO algorithm shows good accuracy in different data sets. This model averages better performance on Tomcat, standard widget toolkits, and Java development tool datasets than other comparison methods. The average reciprocal rank and average accuracy of the weight combination of PSO algorithm are very high. Moreover, this method is greatly affected by the dimension of the word embedding model, and the 300-dimension word embedding model has the best effect. To sum up, this study proposes a good statistical translation model for humanoid robot English translation, which lays the foundation for intelligent interaction between humanoid robots.

## Introduction

Machine translation is a research field that combines artificial intelligence with natural language processing. The purpose is to realize the mutual translation between two natural languages and complete the equivalent conversion of information between different languages on the premise of retaining the meaning of the original text^1^. With the acceleration of globalization, the connection between users of different languages is getting closer and closer, and people’s demand for bilingual interaction and conversion is stronger. In the era of underdeveloped computer technology, the traditional manual translation is inefficient. The emergence of automatic translation technology based on computer technology saves resource waste and improves translation efficiency^2^. Machine translation has become one of the hot topics in the field of applied technology. Machine translation research began in seventeenth century. At the beginning of the research, machine translation mainly adopts the rule-based method, and linguists input the conversion rules between two languages into computers^3^. However, this method is highly dependent on the quality of language rules, and its application is greatly limited^4^. Therefore, a corpus-based translation model, namely statistical machine translation, came into being, which can automatically learn language conversion rules from the corpus^5^. However, this translation method also has limitations, and cannot make good use of global features^6^. With the rapid development of modern science and technology, people pay more and more attention to robot education. In recent years, humanoid robots have gradually entered the classroom teaching. It has been recognized by colleges, teachers, students, and parents. The application of humanoid robot in classroom teaching has effectively improved the efficiency of management teaching, auxiliary teaching and presiding teaching. It can not only stimulate students’ interest and enthusiasm in learning, but also promote the reform of the new curriculum to a certain extent, which is bound to play a powerful boosting role in improving the teaching effect and promoting students’ all-round development. As a new and high technology, humanoid robot integrates artificial intelligence, mechanics, computer, communication, control, machinery, and other disciplines. The rational and scientific application of humanoid robot in education and teaching can not only create a comfortable learning environment for students, but also fully mobilize students’ learning enthusiasm and initiative. This study is developed to explore the translation function of humanoid robot.

Blockchain is the combination of blocks with each other to form a blockchain database. These nodes of the blockchain abide by the exchange protocol together and join the transaction database network of the blockchain. In the traditional data centralized architecture, data is stored centrally, which has great risks caused by the architecture. Blockchain is an embodiment of the decentralization protocol determined according to distributed systems, and builds a distributed architecture with regional units. The information that is calculated and transmitted based on the value exchange protocol is copied to each unit of the entire network. The system determines the selection of information data content through accounting algorithm, and takes time stamp as an important reference, thus realizing distributed database storage based on blockchain. Blockchain is applied in the field of education, which can optimize the educational business process by building an educational trust system, strengthening the protection of intellectual property rights, and realizing efficient and low-cost educational resources. Decentralized education system can be constructed by using the characteristics of decentralization. Distributed storage is adopted to record trusted learning data, and educational intelligence of contract algorithm is developed, so as to build a new mode of network resources and platform operation. The application research of blockchain technology and intelligent learning robot is a frontier issue at home and abroad in recent years.

Deep learning technology can improve current machine translation^7,8^. Firstly, a machine translation framework based on deep learning is constructed, and its language model, word vector, output module, and word alignment are described in detail. Secondly, an English subtitle translation system is designed, and a translation robot automatic question and answer interaction module and a hybrid recommendation mechanism for personalized learning based on blockchain technology are constructed. Finally, sentence embedding and PSO are employed to locate the defects of translation software. The innovation lies in that this work proposes a software defect location method which combines sentence embedding technology and PSO algorithm. In addition, since there is no suitable data set, the system also generates Chinese and English subtitle corpus through crawler technology and corresponding preprocessing process. In short, this model provides a reference for the development of deep learning and blockchain technology in machine translation. It lays the foundation for accurate translation and intelligent interaction between humanoid translation robot and human.

## Material and methods

### Dataset and experiment establishment

The data set based on the deep learning English subtitle translation part is acquired by crawling technology on the YYeTs website^9,10^. The specific method is expounded in the next section. The datasets used in PSO-based software maintenance come from three Java open-source projects, Standard Widget Toolkit (SWT), Java Development Tools (JDT), and Tomcat. The first 300 data are used in the training phase, and 40% of the three open-source projects are used as the validation set and 60% as the training set in the testing phase.

The specific parameters of the experimental equipment used are shown in Table 1.

**Table 1:** Experimental equipment parameters.

Item	CPU	Operating system	Graphics card
Deep learning-based English subtitle translation	Intel Core i7-4790K@4.00ghZ quad core	Windows 7 Ultimate 64-bit SP1	Nvidia GeForce GTX 970(4 GB/Colorful); Python: 2.7; Theano: 0.7; CUDA (Compute Unified Device Architecture): 7.0
PSO-based software maintenance	Inter Coretm I9-7900X; GeForce GTX 2080Ti	Ubuntu 16.04 64-bit	Pycharm 2019.2 IDE; Python: 3.7

### Deep learning-based machine translation

**Figure 1:**
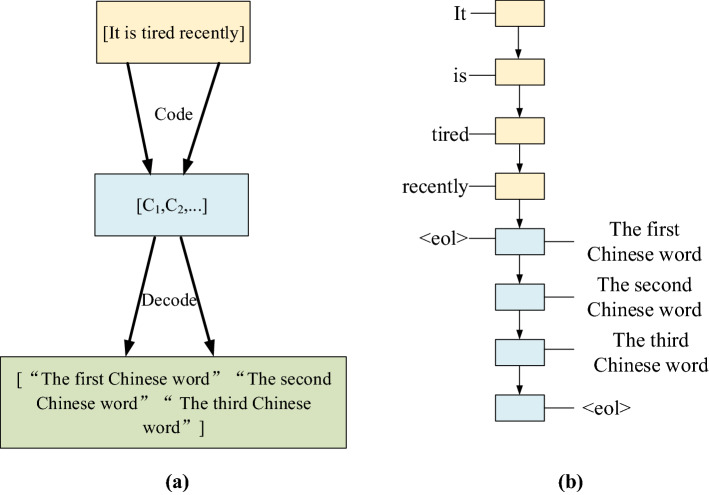
Encoder-decoder framework and time series [(**a**) is the encoder–decoder framework, (**b**) is the word-order time series].

The application scenario of this deep learning is subtitle translation. First, the encoder-decoder framework shown in Fig. 1 is constructed^11^.

In Fig. 1a, the framework first completes the feature expression of the source language sequence, which is realized by encoder learning. Secondly, the corresponding target language sentences are generated by the decoder^12^. The training of the entire model under this framework does not require additional statistics of phrase occurrence probability, word frequency, etc., but only needs to provide corresponding bilingual pairs. The framework only needs to decode part of the network structure and concrete encoding. The order of the input and output sentences in Fig. 1a needs to be executed chronologically, which shows the Chinese translation of a four-word English sentence, where *c* is a fixed-length vector.

In Fig. 1b, each square represents a time, and the translation process of the model will predict the next word and will not stop until the end of the sentence is input. After the translated sentences are input in sequence, the model encodes a fixed-length vector, encodes all the information of the input sequence into the vector, and performs step-by-step prediction of the output sentence in chronological order^13^.

**Figure 2:**
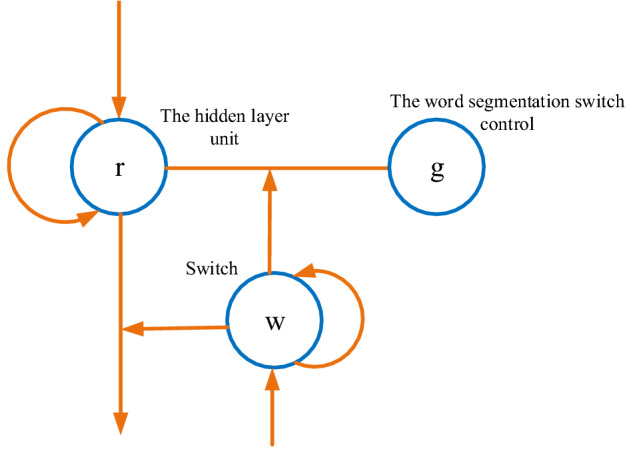
Word vector generation unit.

The above is only the basic framework, and the next step of the network structure is designed. The first is the word vector generation area design. Word vectors can digitize natural language symbols^14^. Word vector generation methods include One-Hot-based methods and distributed representation-based methods^15^. Most of the methods based on distributed representation are at the word level. Of course, the current mainstream natural language processing also uses various word segmentation machines to first cut a sentence into words. In addition to this, however, this research also involves a training method of character data. To optimize the training of such data, a word vector generation method based on RNN is proposed (Fig. 2).

The word vector generation in Fig. 2 is based on each time unit, and it is also expanded in the chronological direction in the input sequence. The hidden layer unit is *r*, and the word segmentation switch control is *g*. Switch* w* clears *g* and *r* at the same time. The generation method of word vector is shown in Eqs. (1)–(5).

1 $${\text{r}}_{\text{i}}= \text{tan} \, {\text{r}}\left({\text{W}}_{\text{h}}{{\text{x}}}_{\text{i}} {+} {\text{U}}_{\text{r}}{{\text{r}}}_{\text{i-1}}\right)$$

2 $$\underline{{{\text{w}}_{{\text{i}}} }} = \sigma \left( {{\text{W}}_{{\text{w}}} {\text{x}}_{{\text{i}}} + {\text{U}}_{{\text{w}}} {\text{h}}_{{{\text{i}} - 1}} + {\text{V}}_{{\text{w}}} {\text{w}}_{{{\text{i}} - 1}} } \right)$$

3 $${\text{w}}_{{\text{i}}} = \left\{ {\begin{array}{*{20}l} {0,} \hfill & {{\text{if}}\;\underline{{{\text{w}}_{{{\text{i,1}}}} }} \ge \underline{{{\text{w}}_{{{\text{i,2}}}} }} } \hfill \\ {1,} \hfill & {{\text{if}}\;\underline{{{\text{w}}_{{{\text{i,1}}}} }} < \underline{{{\text{w}}_{{{\text{i,2}}}} }} } \hfill \\ \end{array} } \right.$$

4 $${\text{g}}_{\text{i}}^{\text{x}} {=} {\text{w}}_{\text{i}}{{\text{r}}}_{\text{i}}$$

5 $${\text{r}}_{\text{i}} {=} \left(\text{1} - {\text{w}}_{\text{i}}\right){\text{r}}_{\text{i}}$$

In Eqs. (1)–(5), σ is the sigmoid activation function. In Eq. (1), *r*_*i*_ is the output of the original hidden layer. In Eq. (2), *w*_*i*_ is used to determine whether *r*_*i*_ at time *i* is output. Equation (5) is used for further updating of *r*_*i*_. All equations omit the bias term in order to reduce the confusion of the equation.

The encoder part uses a convolutional neural network, while the decoder part uses an RNN. For the encoder part, an output gate and two control gates are added based on the convolutional neural network. In the decoder language model, a vector containing the coding module information is added based on the RNN, and the vector belongs to a source language information. The word alignment module generation method adopts the “encoding–decoding” mode^16^. The word alignment step is added, and the encoding module information is integrated by adding a vector. The vector here is also expanded into two vectors according to the order of target sentence generation. The output module belongs to the correction module, which is embedded in the decoding part and needs to further output the translated words. Therefore, the output module must combine the previous output word again, decode the previous state of part of the hidden layer, and translate the required source language information.

## English subtitle translation system design

The used data belongs to the bilingual Chinese and English in the spoken language type, and the existing data is relatively small, so the crawler technology is adopted to collect the relevant data. The YYeTs website in the movie subtitle resource is used as the crawling object, and the subtitles of this website are more authoritative.

First, the data is located, and the pages that need to be crawled are manually observed. The subtitle web page is loaded, and the movie page link is extracted. Secondly, the Hyper Text Markup Language (HTML) source code of the individual movie subtitle page is analyzed, and the Chinese subtitle compressed file download link of the corresponding page is extracted. To ensure the complete extraction of the link information in the first two steps, it is necessary to carry out a step-by-step hierarchical screening of the required tags according to the HTML tags combined with Extensible Markup Language Path Language (XPath)^17^. Through the crawler program, all Chinese and English bilingual subtitle compressed packages on the YYeTs website are harvested, totalling 16,986. Due to the low download efficiency of the single-threaded crawler system, it takes a week to download all the data. Therefore, the multi-threaded parallel processing is performed. First, the original task is overwritten to get a subclass that inherits from the Thread class, and the thread pool is opened. The original task consists of four sub-tasks, namely, obtaining a list of movie links, a list of subtitle links, a list of compressed file links, and a letter compressed file. The four subtasks run in multiple threads, the thread pool size is 20, and a single thread waits for processing between the subtasks. The time for the crawler system to obtain data after multi-threading is one day.

Data pre-processing mainly refers to the structured processing of data. First, the compressed files with the suffixes of zip and Roshal A Rchive (RAR) in the downloaded files are decompressed, and two types of subtitle files, Advanced Sub Station Alpha (ASSA) and Sub Rip Text (SRT) are obtained^18^. These two kinds of files are small, and the modification to them can be handled by using the system’s own Notepad. Finally, the bilingual pairs with chronological order are harvested, monolingual files are filtered, and the selected documents are transcoded to 8-bit Unicode Transformation Format (UTF-8). A total of 13,717,370 Chinese and English bilingual corpora are obtained and stored in the *zh.pkl* file and the *en.pkl* file. Due to the different serialization requirements at different levels, data pre-processing adopts two processing methods, namely word-level and character-level.

The data structure processing adopts the current common processing method. The word-level dictionary retains the first 30,000 words with high frequency, and the number of character levels is small, which is used directly. For the expression of text, the One-Hot encoding is adopted. To optimize the training efficiency, the task processing is optimized by sorting sentences by sentence length at regular intervals, and a small block is synthesized nearby for task processing^19^. The above data is compressed and stored by the Hierarchical Data Format (HDF) algorithm.

The parameters in model training are set to 2000-word lengths in the language model. The word embedding module is finally compressed into a low-dimensional vector with a dimension of 1000. The word-aligned part vector size is 2000, and the length of the output module is 1000. During the experiment, a series of weight matrices in the recursive mode are initialized. The random orthogonal matrix can be randomly generated first and then obtained by the singular value decomposition method^20^. The matrices in the alignment module are randomly sampled using a Gaussian distribution with a variance of 0.0012 and a mean of 0. The bias vector of the weight matrix is 0. The weight matrix is randomly sampled from a Gaussian distribution with a variance of 0.0012 and a mean of 0.

The parameter training adopts the Mini-Barch method of the Stochastic Gradient Descent (SGD) algorithm. One gradient update of this method is shown in Eqs. (6) and (7).

6 $${\text{x}}_{{\rm t}+1} {=} {\text{x}}_{\text{t}}{+ \Delta }{\text{x}}_{\text{t}}$$

7 $$\Delta {\text{x}}_{\text{t}}{=-\delta} \cdot {\varepsilon}_{\text{t}}$$

In Eq. (6), *x* is the parameter to be trained, and *t* is the time sequence. In Eq. (7), δ represents the gradient, and ε is the learning rate. The automatic adjustment of the parameter learning rate in the experimental part is carried out by the AdaDelta algorithm. The calculations are shown in Eqs. (8)–(10).

8 $$\Delta {\text{x}}_{\text{t}}{=-}\frac{{{\rm RMS}[\Delta x}{]}_{\text{t-1}}}{\text{RMS}{[\varepsilon]}_{\text{t}}}{\cdot}{\text{g}}_{\text{t}}$$

9 $$\text{RMS}{[\varepsilon]}_{\text{t}}{=}\sqrt{{\text{E}}{\left[{\varepsilon}^{2}\right]}_{\text{t}}{+\epsilon}}$$

10 $${\text{E}}{\left[{\varepsilon}^{2}\right]}_{\text{t}}{=} \rho \text{E}{\left[{\varepsilon}^{2}\right]}_{\text{t}-1}{+(1-\rho)}{\varepsilon}_{\text{t}}^{2}$$

Equation (9) is the root mean square, and Eq. (10) is a similar approach to the momentum factor averaging algorithm.

The recursive part of the network is processed by the Backpropagation Through Time (BPTT) algorithm. The evaluation index of model translation quality is shown in Eq. (11).

11 $${\text{BLEU}} = \xi \times {\text{exp}}\left( {\sum\limits_{{{\text{n}} = 1}}^{{\text{N}}} {\left( {{\text{w}}_{{\text{n}}} {\text{logP}}_{{\text{n}}} } \right)} } \right)$$

BLEU in Eq. (11) is Bilingual Evaluation Understudy. *w* is the weight, ξ is the penalty factor, and *P* is the accuracy. Two million pairs of bilingual data are selected as experimental data, and 1.8 million pairs of training set and 5000 sentences of test set are generated. The sequence length, model score, model translation comparison, etc. will be generated in the later part.

## Translation software maintenance of sentence embedding collaborative PSO

**Figure 3:**
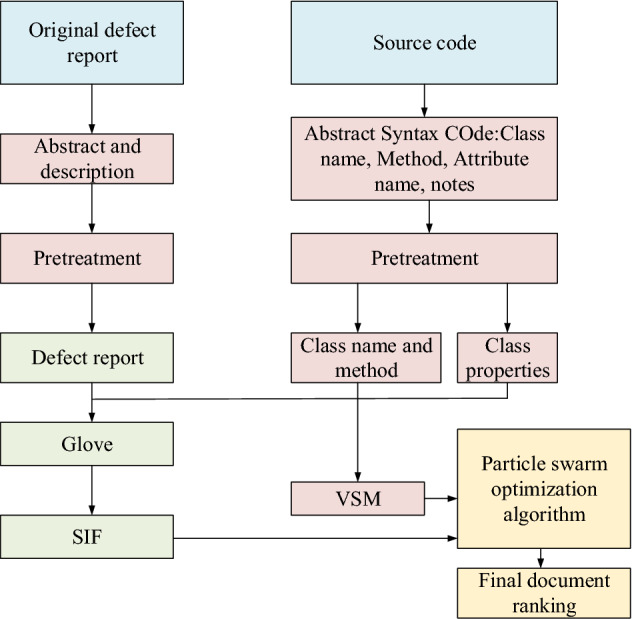
Software defect location process.

This section is maintained for meta-heuristic-based translation software. The structure of the software defect location method used is shown in Fig. 3.

Figure 3 shows that the software defect location process mainly includes extracting source code content with abstract syntax tree technology and preprocessing defect report with natural language technology. The semantic similarity between the source code and the software defect report is calculated, and the text information quantification is combined with the Vector Space Model (VSM) technology. The PSO algorithm is employed to sort the most weighted combination^21^.

The data pre-processing of the software defect location part includes the description and summary of the defect report. First, it creates an abstract syntax tree, tokenizes text with spaces, and removes numbers, punctuation, and stop words. Second, it splits the compound words. Finally, it stems all words, using tools in the Natural Language Toolkit (NLTK) package here.

Smooth Inverse Frequency (SIF) algorithm and word embedding technology are adopted to calculate the semantic similarity between software defect reports and source code files. The first is word vectorization, but the SIF model can only input vectorized words^22^. Descriptions, summaries, and source code files in software bug reports are converted into vectors using Glove word embedding techniques^23^. The second is word probability calculation. In SIF, the probability of words in a sentence needs to be calculated, as shown in Eq. (12)^24^.

12 $${\text{P}}({\text{w}}| {\text{c}}_{\text{s}}){=} \gamma \text{p} (\text{w})+(1-\gamma) \frac{{\text{exp}}\left({<}{\widetilde{\text{c}}}_{\text{s}}{,}{\text{v}}_{\text{w}}{>}\right)}{{\text{Z}}_{{\widetilde{\text{c}}}_{\text{s}}}}$$

In Eq. (12), γ is the scalar hyperparameter, ${\widetilde{\text{c}}}_{\text{s}}$ is the vector representation of the sentence, and *v*_*w*_ is the word semantic vector.

The word embedding calculation is shown in Eqs. (13) and (14).

13 $${\text{P}}\left({\text{s}} \vert {\text{c}}_{\text{s}}\right){=}\prod_{{\text{w}} \in {\text{s}}} \,\, {\text{P}}\left({\text{w}}\vert {\text{c}}_{\text{s}}\right){=}\prod_{{\text{w}} \in {\text{s}}} \,\, \left[\gamma \text{p(w)}\vert {+(1- \gamma )}\frac{{\text{exp}}\left({<}{\widetilde{\text{c}}}_{\text{s}},{\text{v}}_{\text{w}}{>}\right)}{{\text{Z}}_{{\widetilde{\text{c}}}_{\text{s}}}}\right]$$

14 $${\text{argmax}}\sum_{{\text{w}} \in {\text{s}}} \,\, {\text{f}}_{\text{w}}\left({\widetilde{\text{c}}}_{\text{s}}\right) \propto \sum_{{\text{w}} \in {\text{s}}} \,\, \frac{\text{a}}{\text{P(w)+a}}{\text{v}}_{\text{w}}{,} \, \text{a} = \frac{1- \gamma }{ \gamma\text{Z}}$$

Equation (14) is the maximum likelihood estimate of the unit sphere in Eq. (13).

The SIF word weighting factor is set to 0.00001, the number of singular vectors is 1, and the dimension of the Glove model input into the SIF model is 300.

The surface lexical similarity is calculated using VSM, as shown in Eq. (15).

15 $$\text{TF-ID}{\text{F}}_{\text{i,j}}= \text{T} {\text{F}}_{\text{ij}}\text{*ID}{\text{F}}_{\text{j}}$$

In Eq. (15), $\text{TF-ID}{\text{F}}_{\text{i,j}}$ is the weight of the *j*th word in the *i*th document, *TF* is the frequency, and *n* represents the number of documents, where ${\text{ID}}{\text{F}}_{\text{j}}\text{=}\frac{\text{n}}{{\text{D}}{\text{F}}_{\text{j}}}$.

The final obtained surface vocabulary similarity is shown in Eq. (16).

16 $$\text{sim(br,sr)}= {\rm max} (\text{cos(pair)}{|}{\text{m}}_{\text{j}} \in {\text{sr}})$$

In Eq. (16), *br* represents a software defect report, and *sr* represents a source code file.

The PSO algorithm-based optimal combination calculation of linear weights is shown in Eq. (17).

$${\text{finalscore}} \, ={\text{w}}_{1}\cdot \text{SIFScore }+{\text{w}}_{2}\cdot {\text{VSMScore}}+{\text{w}}_{3}\cdot {\text{RecencyScore}}+{\text{w}}_{4}.$$

17 $${\text{FrequencyScore}}{+}{\text{w}}_{5} \cdot {\text{CollaborativeFilteringScore}}$$

In Eq. (17), *w*_1_ is the similarity weight calculated by the SIF model, *w*_2_ is the cosine similarity obtained by the term frequency–inverse document frequency (TF-IDF) model, *w*_3_ is the defect repair record weight, *w*_4_ is the defect repair frequency weight, and *w*_5_ is the collaborative filtering score weight. SIFScore refers to the score using the SIF source code file. VSMScore refers to the score using the TF-IDF model. RecencyScore refers to the defect repair score. FrequencyScore refers to the defect repair frequency score. CollaborativeFilteringScore refers to the collaborative filtering scores. The weight is calculated using the PSO algorithm, and the calculation of particle position and velocity is shown in Eq. (18).

18 $${\text{v}}_{\text{id}}^{\text{t}+1}= \text{w} {\text{v}}_{\text{id}}^{\text{t}}{+}{\text{c}}_{1}{\text{rand}}\left({\text{p}}_{\text{id}}^{\text{t}}-{\text{x}}_{\text{id}}^{\text{t}}\right){+}{\text{c}}_{2}{\text{Rand}}\left({\text{p}}_{\text{gd}}^{\text{t}}-{\text{x}}_{\text{id}}^{\text{t}}\right)$$

In Eq. (18), *w* is the inertia weight, and *c* is the learning factor. *rand* is a random value, where, ${\text{x}}_{\text{id}}^{\text{t+1}}{=}{\text{x}}_{\text{id}}^{\text{t}}{+}{\text{v}}_{\text{id}}^{\text{t}+1}$.

The evaluation metrics used in this experimental evaluation are Accuracy@k, Mean Average Precision (MAP), and Mean Reciprocal Rank (MRR). Accuracy@k is the percentage of software bug reports that match at least one correct suggestion in the top *k* files. The specific calculations of the MAP and MRR are shown in Eq. (19) and Eq. (20), respectively.

19 $$\text{MAP} = \frac{1}{{\text{N}}}\sum_{\text{i}=1}^{\text{N}}\,\,{\text{AvgP}}{(}{\text{i}}{)}$$

In Eq. (19), *N* is the number of source code files, where, ${\text{AvgP(i)}}= \sum_{\text{j=1}}^{\text{M}} \,\, \frac{\frac{\text{Q(j)}}{\text{j}}\text{*bool(j)}}{\text{B(i)}}$. *M* is the number of software defect reports. *B* is the number of defect files included in the software defect report. *bool(j)* indicates whether the source code file ranked *j* is a defect file. *Q* indicates the number of source files related to the defect report in the source file.

20 $${\text{MRR}} = \frac{1}{{\rm{N}}}\sum_{\text{i}=1}^{\text{N}} \,\, \frac{1}{{\text{ first }}_{\text{i}}}$$

In Eq. (20), *N* is the number of defect reports, and *first* is the file location where the first defect is located.

The parameters for this experiment are set as the population size of 20, the inertia weight of 0.8, the learning factors of 1 and 2, and the maximum number of iterations of 1000.

The results in the following part include the performance of the proposed method and the benchmark method on different data sets. The compared models include Deep Neural Networks Loc (DNNLoc), BugLocator, and Learning-to-Rank. Validity test of PSO algorithm and comparison of embedding effect of words of different dimensions are also performed.

## Construction of automatic question answering interactive module for the translation robot

On the basis of the above accurate translation, an intelligent interaction module of the robot is designed, and a humanoid translation robot with translation and interaction functions is obtained. Convolutional neural network has been applied in classification, pattern recognition and other fields with its powerful learning ability. The method achieves feature extraction based on convolution, and classifies on this basis to achieve higher classification accuracy. The network is consisted of convolution, pooling, and full connection layers. Given that the two sentences will be analyzed and classified, the structure of parallel convolutional neural network is adopted. ReLU function is taken as activation function. To avoid the overfitting problem, the dropout method is introduced to modify the convolutional neural network. AdaGrad adaptive learning algorithm is selected for learning rate. This module can realize the intelligent learning, classification, and answer of dialogues.

Although convolutional neural network can realize the extraction of deep features, there are still defects in the application, especially in the case of massive statements, and it is difficult to retain the underlying semantic information. To solve these problems, attention mechanism is introduced. Traditional Neural Machine Translation (NMT) includes encoder and decoder, which can get target language through decoding. Combined with attention mechanism, it is introduced into NMT neural machine translation. The whole system is divided into multiple modules. Each module has different functions and structures and achieves the overall function depending on the connection and coordination between each part. Question and answer engine is mainly used to process the input information. The semantic matching part is the algorithm based on the convolutional neural network constructed in this study.

Humanoid robot is a kind of high technology, which integrates artificial intelligence, mechanics, computer, communication, control, machinery, and other disciplines. The reasonable and scientific application of humanoid robot in the process of education and teaching can not only create a comfortable learning environment for students, but also fully mobilize their learning enthusiasm and initiative. At present, the teaching of humanoid robots is mostly carried out in the form of groups, which requires the members of the groups to work together and use the collective strength to solve problems that are difficult for individuals. By studying in this form, students can easily improve their teamwork ability. The reasonable application of humanoid robot in education and teaching can popularize more robot knowledge to students. Robot classroom teaching has become the core content of information technology education in colleges and universities. Robots are no longer mysterious, but have entered students’ daily study and life, which is of great help to students’ sustainable development. Therefore, the automatic question-and-answer interactive module of translation robot in this work is added to the production of humanoid robots to increase their teaching ability.

## Hybrid recommendation mechanism based on personalized learning

The system of blockchain technology and intelligent humanoid learning robot presents a distributed structure. The nodes of this structure include family, school, community, venue, training class, social welfare home, public place, and travel. The central node is family and school. The main functions of the node are block creation, recording, transmission, and encryption. The family node records its learning process in the Block. After confirmation, the system sends the learning record chain to all nodes. Over and over again, every node in the system keeps a uniform record. The intelligent learning service system records the learning process by creating blocks. The pre-recorded contents of each data block are magic number, block size mark, data block header information, learning activity count, and learning activity information. Where, the calculated value of the data block header information is the reference target number of the calculated value of the next new block, and the last learning activity details records all learning activities in this block.

The hybrid recommendation mechanism based on personalized learning is the fusion of collaborative filtering recommendation technology, content-based recommendation technology and knowledge-based recommendation technology. Collaborative filtering recommendation uses the historical behaviors or opinions of existing learners to predict the most recommended learning resources for current learners. The input information is the evaluation data of the resource rating matrix, and the output data is the predicted value of the learner’s preference for this resource and a list of multiple recommended resources.

Content-based recommendation is a classic dynamic recommendation idea, which matches resource characteristics and learner preferences. The recommendation focus mainly includes two aspects: on the one hand, dynamic generation based on historical records. Although this recommendation method relies on additional information about resources and learner preferences, it has the advantage of generating recommendation lists in the absence of learner group information. Content-based recommendation mainly includes content similarity-based retrieval and probability-based methods, while knowledge-based recommendation is a recommendation method formed by clarifying recommendation rules in the form or basis of the similarity between learners’ needs and resources. Including constraint-based recommendation and instance-based recommendation. The difference lies in that the constraint-based recommender system relies on a well-defined set of recommendation rules, and it searches for the set of resources to be recommended among all resource sets that meet the recommendation rules.

In addition, content-based recommendations include a big data approach, supported by large-scale learning services and blockchain technology. For example, different learning state transition graphs are formed through the matching of relevant learner trajectories, or the dynamic programming method of closed-loop dynamic learning data information collection is carried out through the self-quantization learning process of big data. The characteristics of learning resources pushed by the system are accuracy, comprehensiveness, and personalization. Learners can quickly obtain this information, thus saving the time of searching and finding materials on the Internet. Personalized recommendation is the core of learning resource push. The system obtains the learner’s specific learning situation from the learning management database, so as to screen out the relevant information that best matches the learner’s knowledge level.

## Results and discussion

### Result analysis of deep learning-based English subtitle translation system

The statistical results of different language lengths at the word level and character level are shown in Table 2.

**Table 2:** Language length statistics.

Length	Word level		Character level	
	Train/en	Test/zh	Train/en	Test/zh
1–9	1,270,522	1,366,950	97,169	873,449
10–19	520,850	432,225	256,970	904,566
20–29	4921	820	417,398	21,902
30–39	3137	5	456,448	78
40–49	541	–	339,007	3
50–59	29	–	181,520	2

Table 2 shows that the statistical results are divided into training data and test data. In terms of word-level and character-level comparisons, character-level modeling is more challenging.

**Figure 4:**
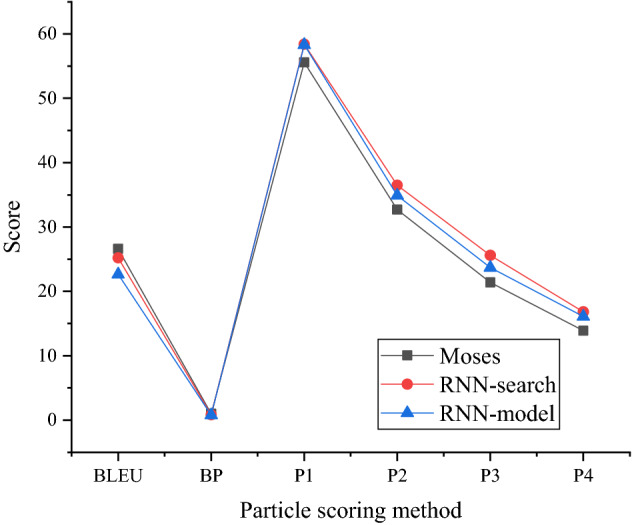
Scoring results of different word-level translation models.

**Figure 5:**
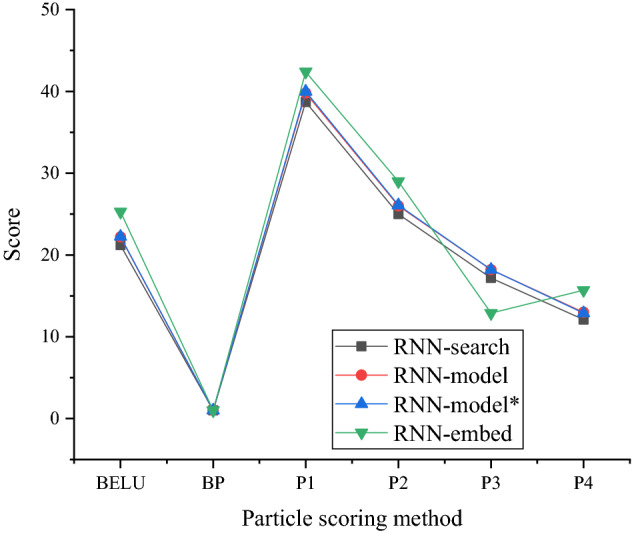
Scoring results of different character-level translation models.

The scoring results of different translation models are shown in Figs. 4 and 5.

The comparison model shown in Fig. 4 is the Moses model, and Recurrent Neural Network (RNN)-search is the current optimal neural network-based method. P1-P4 in the abscissa show the matching accuracy. In Fig. 4, the difference of scores of the three models is relatively small, and the score of the RNN-model is basically between the Moses model and the RNN-search model. The word-level RNN-model scores does not improve. See the “drawing data” table in the supplementary information for the drawing data of all figures in this paper.

In Fig. 5, there are many contrasting models due to the challenging character-level modeling. The asterisk represents the improved word vector generation algorithm of RNN, and RNN-embed is an RNN-based word embedding model. The curve in Fig. 5 shows that RNN-embed has superior performance in the rest of the evaluation results except for the slightly lower accuracy at point P3. The RNN-embed algorithm has a certain word clustering effect.

Table 3 shows the parameter tuning process of the RNN-embed model in Figs. 4 and 5.

**Table 3:** The parameter adjustment process of the combined model.

Parameter type	Number of parameter adjustments			
	1	2	3	4
Longest sentence threshold	50	80	100	80
Dim	1000	1000	2000	2000
Rank_n_approx	620	1000	1000	1000
Pn	39.9/26.4/18.9/14.0	40.8/27.3/19.7/14.7	40.7/27.4/19.8/14.8	42.4/29.0/21.2/15.7

Table 3 shows that the RNN-embed model has been significantly improved after the fourth parameter adjustment. At that time, the model is not overfitted, the results of the training model are improved, and the single process is slow.

Based on the previous content, the performance of the RNN-search model and the RNN-embed model is relatively better, so the actual translation capabilities of the two models are compared, and the comparison translation method also includes Baidu translation. Since the data used are movie subtitles, there are certain requirements for the spoken translation of the model. Examples of translations for different translation methods are shown in Table 4.

**Table 4:** Translation examples of different translation methods.

The original text	May I have a look at this picture?	I love you
RNN-search	May I see this photo?	I like you
RNN-embed	May I have a look at this picture?	I love you so much
Baidu translator	Can I see this photo?	I love you
Reference	May I see this photo?	I love you

In Table 4, the example sentences are common colloquial phrases, the training data of the two models is at the character level, and the training process of Baidu Translate is at the word level. Due to the addition of word embedding improvements, the RNN-embed model learns a more colloquial intonation.

**Figure 6:**
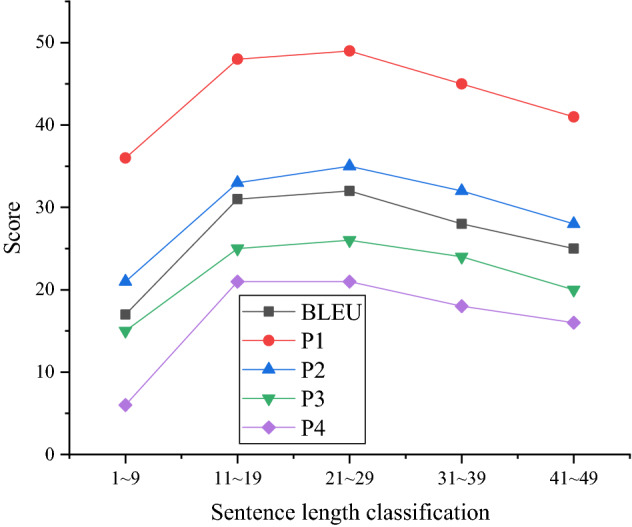
Test results for sentences with 1–49 words in length.

**Figure 7:**
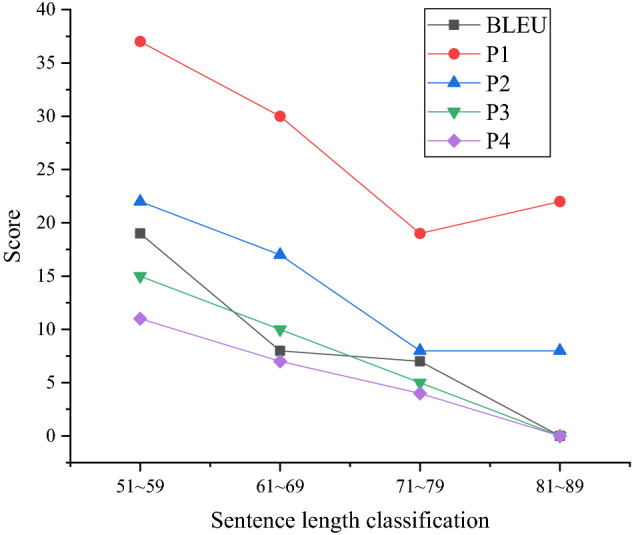
Test results for sentences with 51–89 words in length.

Figures 6 and 7 are comparisons of test results classified regarding sentence length. Among them, the average length of character-level data is 32, and the average length of word-level data is 7.

In Figs. 6 and 7, the curves show a clear downward trend, indicating that the RNN-embed model has a strong ability to process short sentences. The sentences with the strongest translation outcome are between 11 and 39 words. The sentences with the weakest translation outcome are 71–79 words in length. Hence, the model needs to strengthen the processing of long dramas, especially character-level input, and the average sentence length is much larger than that of word-level input.

### Analysis of maintenance results of PSO-based translation software

**Figure 8:**
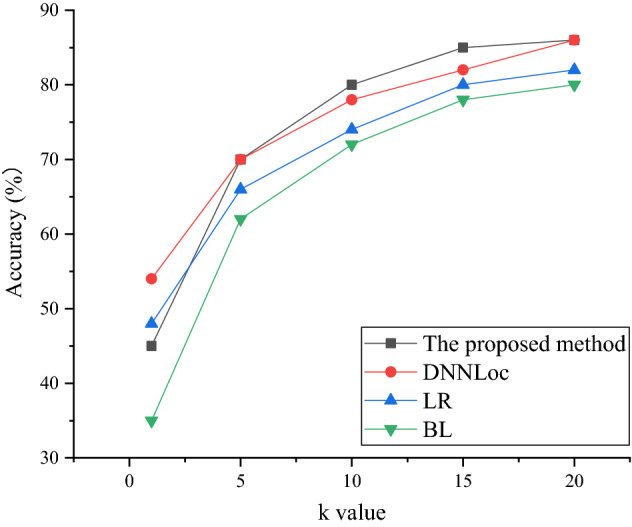
The accuracy on the Tomcat dataset.

The Accuracy@k metric results of different models on the Tomcat dataset are shown in Fig. 8.

**Figure 9:**
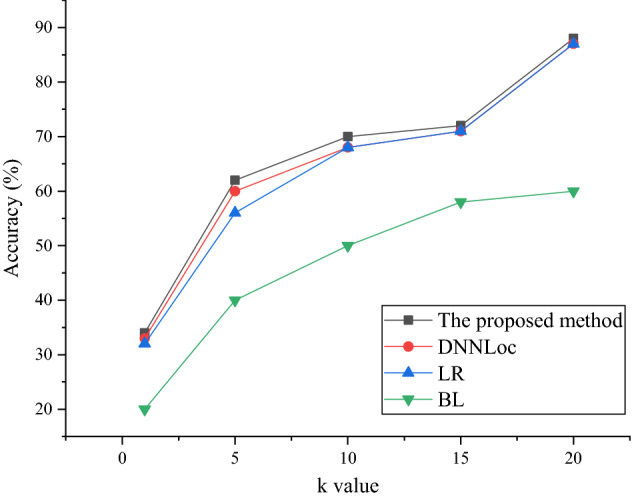
The accuracy on the JDT dataset.

**Figure 10:**
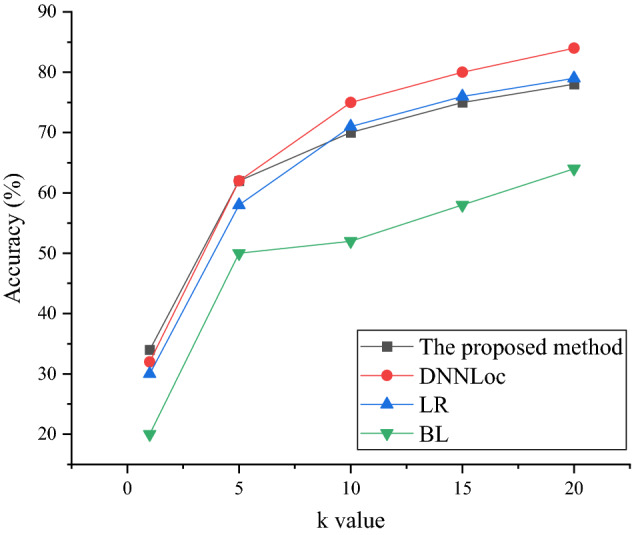
The accuracy on the SWT dataset.

Figures 8 and 9 show that the proposed method shows an excellent accuracy on both the Tomcat dataset and the JDT dataset, which is better than other models. In Fig. 9, when the number of source code files *k* is 0, the accuracy of the proposed method is 34%. When there are 15 source code files, the accuracy reaches 73%. The BugLocator model has the lowest overall accuracy. The accuracy of the DNNLoc model and the Learning-to-Rank model is good. In Fig. 10, the average accuracy of the proposed method is basically the same as that of the Learning-to-Rank model.

**Figure 11:**
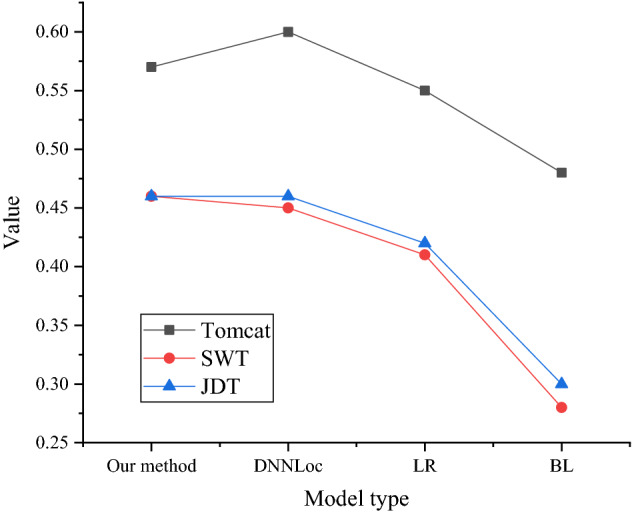
MRR on different datasets.

**Figure 12:**
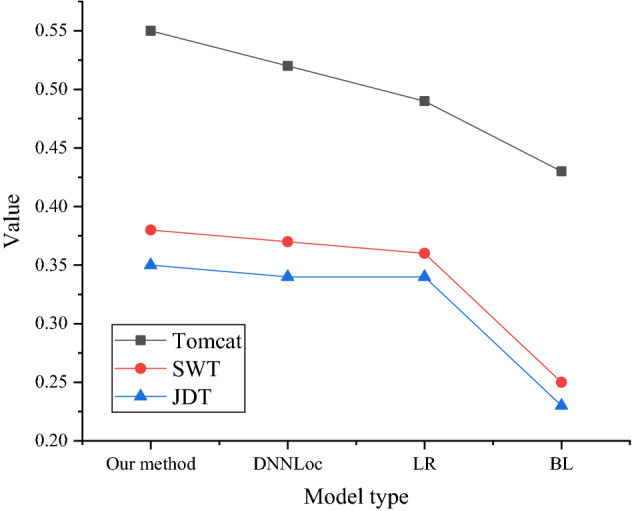
MAP on different datasets.

The MRR and MAP results on different datasets are shown in Figs. 11 and 12.

Figure 11 shows that the proposed model basically presents good results. The MRR of the proposed model on the Tomcat dataset is 0.54, that of the DNNLoc model is 0.6, that of the LR model is 0.55, and that of the BL model is 0.5. Only on the Tomcat dataset, the MRR of the proposed model is slightly lower than that of the DNNLoc model. On the SWT dataset and the JDT dataset, the MRR of the proposed model is the highest.

In the comparison of MAP on different datasets illustrated in Fig. 12, which is basically consistent with the results presented in Fig. 11, whether it is on the Tomcat dataset, SWT dataset, or JDT dataset, the MAP of the proposed model is the highest. The average effect of the proposed model is better than other comparison methods on the Tomcat, SWT, and JDT datasets.

**Figure 13:**
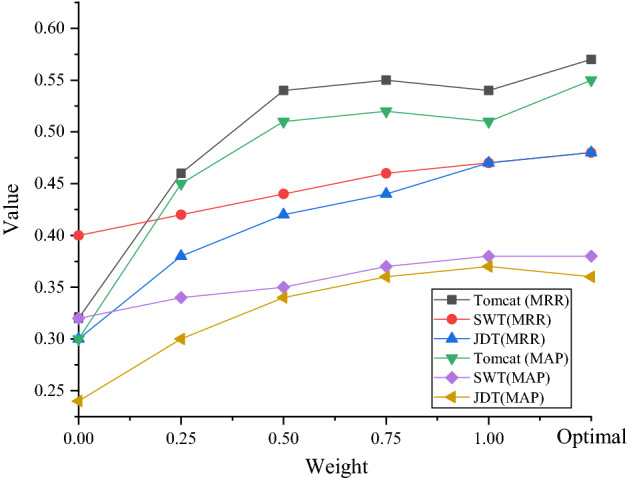
Indicator changes under different w2.

Under different w2 weights, the changes of MRR and MAP are shown in Fig. 13.

In Fig. 13, the abscissa is the five w2 values set in this test to calculate the weight comprehensive score, and the last one is the weight combination calculated by the PSO algorithm. Figure 13 shows that the MRR and MAP of the weight combination of the PSO algorithm are basically high.

**Figure 14:**
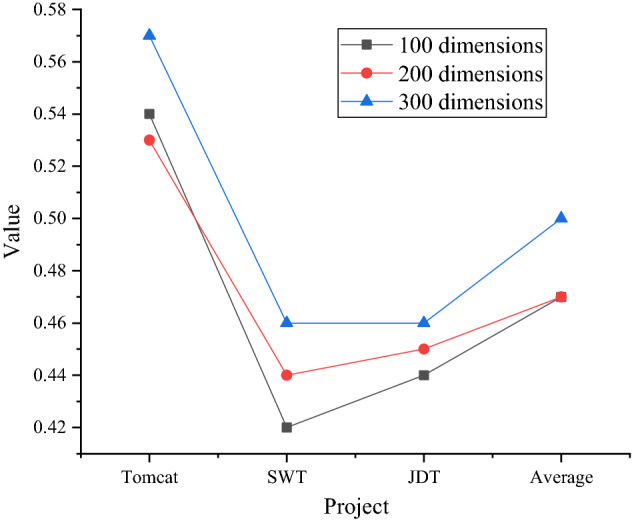
MRR of embedded words of different dimensions.

**Figure 15:**
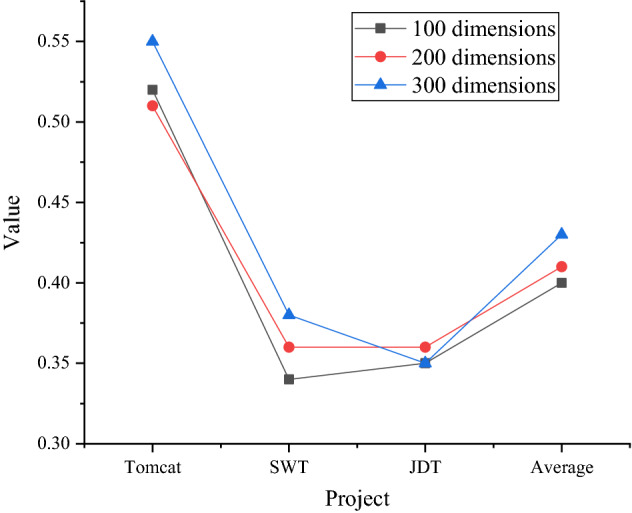
MAP of embedded words of different dimensions.

The embedding effects of Glove words of different dimensions are shown in Figs. 14 and 15.

Figure 14 shows that the proposed method works best in the 300-dimensional word embedding model. The average MRR is high, which is an improvement of 0.03 compared with 100-dimensional and 200-dimensional word. From an average point of view, the average metric values for the 100-dimension and 200-dimension in the graph are the same. The MRR of this method in the 300-dimensional word embedding model reaches 0.5.

Figure 15 shows that the MAP of the proposed method also shows a relatively better effect in the 300-dimensional word embedding model. The MAP of 300-dimension word is 0.03 and 0.02 higher than that of 100-dimension and 200-dimension, respectively. The average MAP of the 200-dimension model is higher than that of the 100-dimension model, and the MAP of the proposed method in the 300-dimension word embedding model reaches 0.43. In Figs. 14 and 15, the proposed method is greatly affected by the dimension of the word embedding model, and the 300-dimensional word embedding model has the best effect.

## Conclusions

The statistical translation model software is designed and optimized based on the application of improved deep learning techniques and meta-heuristic algorithms in the statistical translation model. A translation model is designed by combining RNN and word vector generation algorithm. Combined with Chinese and English subtitle data, the model is trained and its performance is compared. In addition, a software defect location method combining sentence embedding technique and PSO algorithm is proposed. The method uses Glove and SIF to calculate the semantic similarity between software defect reports and source code files. In addition, a question-and-answer interaction module is designed for humanoid translation robot, and a personalized hybrid recommendation mechanism is built based on blockchain technology. Finally, the performance of the model is tested. The results show that the English subtitle translation model and software defect location model have good performance. This work provides a reference for the further development of machine learning and data mining. Applying the results of this work to the humanoid translation robot is expected to achieve a good educational effect in college film majors. Nevertheless, the research still has some shortcomings. The performance of the translation model does not exceed the Moses algorithm, and there is a lexical gap between the software defect report in the software defect location model and the source code file. In addition, the question-and-answer module of humanoid robot is designed, but the algorithm performance of this part is not evaluated. There is also a lack of case studies applying blockchain technology. Therefore, the RNN-based word embedding algorithm will be improved in the future to extract keywords from the text. It should also increase the algorithm performance evaluation for humanoid robot interactive question and answer module and increase the application of blockchain technology. After further improvement, we hope to get a more feasible improvement scheme of humanoid translation robot (Supplementary Information).

## Supplementary Information

Supplementary Information.

## Data Availability

All data generated or analysed during this study are included in this published article [and its supplementary information files].
